# The Times

**Published:** 1862-11

**Authors:** 


**THE TIMES.**—If in this exigency, Omnipotence were to say, "What is thy request?" I should be afraid to utter more than seven monosyllables:

"Thy will be done, make mine thine."

I want no more victories; that implies death to many. I do not want peace, unless it removes the cause of the war, else similar wars would inevitably follow My greatest solace is, "God reigns," and "shall not the Judge of all the earth do right?" If I had my way, human slavery should cease on the instant, but I am not certain that it would be wisest and best, hence I leave the question to the decision of Almighty power, and wisdom, and beneficence, desiring that I may be led to agree thereto. I am not ashamed to have been born and bred in the South. Grander models of true manhood and womanhood are not found elsewhere on earth. They look to God in prayer with as much sincerity as the North. They are mistaken. They have been misled. Our universal prayer should be for them, as our brothers, that God would show them their error. I know that the North is right in the great object of the war, for Omnipotence has shown his approval by favoring the North in the two most important means under his sole control. Such a succession of fruitful seasons has *never* occurred before. Such an exemption from yellow fever and other Southern epidemics, even when Northern troops had to remain South, has not been recorded in our history. Therefore God is with the North. Where he had to work with human agencies, he could only show his favor indirectly; rather by overruling; consequently we have had reverses, but these have been overruled to great good ends. Had Sumter never have fallen, the North would never have been united together as one man. Had we afterwards marched steadily on to victory, Congress would not have passed the confiscation act; and but for the Chickahominy and subsequent misventures, the President would not have felt compelled to issue the emancipation proclamation; and there is reason to fear that victory after victory now, would procure a most unfortunate modification of that document. The raid upon the Border States would appear discouraging; but it will do more than any thing else to cause the people to stand by the government, even if the cherished institution should have to be heaved overboard. And here let me suggest to all praying people, that the agency which they should invoke should be the going out from the Almighty of a spiritual influence over the whole South, and North also, to bring order out of confusion, as when chaos reigned. It is as easy for Omnipotence to change the mind of the South as to give victory to Northern arms; then why not all join in the earnest, hourly prayer, that the Lord would give our Southern brethren a better mind; that he would change their views of things, and help them to see that there is no better government on earth than ours, and that there is no wiser policy than that which relieves us of slavery. It is a great solicitude to me, lest the Faith of Southern Christians should fail, when they see that they can not succeed, but must come back under the laws of the Union, the laws which they helped to make. I hope when these truths do break in upon them, they will still feel like Job, "Though he slay me, yet will I trust in him," and that they may in such faith live long enough to see that God's ways are best; and that like Job, they may in the end have in material wealth, even "twice as much as they had before." Such I believe will be the condition of the whole United States within five years after slavery has ceased utterly.

